#  Fast Electrocatalytic Determination of Methimazole at an Activated Glassy Carbon Electrode

**Published:** 2016

**Authors:** Fahimeh Jalali, Zahra Hatami

**Affiliations:** *Department of Chemistry, Faculty of Science, Razi University, 67346, Kermanshah, Iran. *

**Keywords:** Methimazole, Pretreated glassy carbon electrode, Square wave voltammetry, Tablets

## Abstract

A fast and simple voltammetric method for the determination of methimazole in pharmaceutical products was reported. A glassy carbon electrode was pretreated by anodization at +1.75 V (vs. SCE) for 5 min, followed by potential cycling in the range of 0.3-1.3 V (20 cycles). The pretreated electrode showed an excellent electrocatalytic effect on the oxidation of methimazole. Compared with untreated electrode, a large decrease (~300 mV) in the oxidation peak of methimazole was observed. The oxidation peak current at the new potential (0.4 V vs. SCE) was linearly dependent on the concentration of methimazole in the range of 7.0 - 130 μM with a detection limit of 3.7 μM (S/N = 3). The method was successfully used in the determination of methimazole in thyramozol tablets. Due to the simple and fast electrode preparation, there is no need for electrode cleaning or storage.

## Introduction

**Scheme 1:**
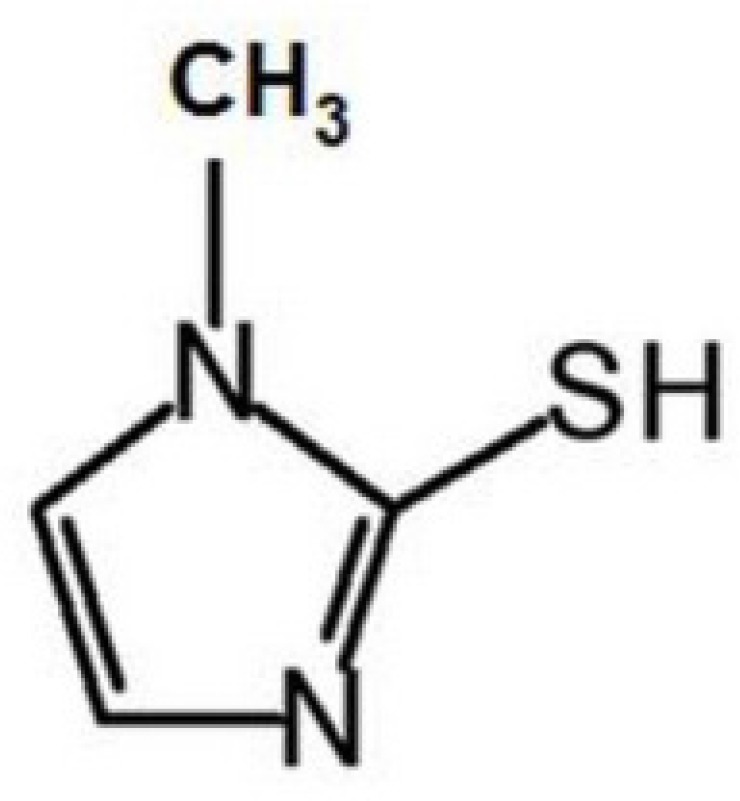
Chemical structure of methimazole

Methimazole (2-mercapto-1-methyl imidazole, MMI) (Scheme 1), is used to treat overactive thyroid (hyperthyroidism). It is also taken before thyroid surgery or radioactive iodine therapy. Grave›s disease is the most common cause of hyperthyroidism. It is an autoimmune disease resulting from antibodies that attach to receptors on thyroid hormone-producing cells in the thyroid gland and trigger over production of thyroid hormone. An enzyme (peroxidase) produces thyroid hormones, i.e., thyroxine (T4) and triiodothyronine (T3), by combining iodine with a protein called thyroglobulin. MMI prevents iodine and peroxidase from their normal interactions with thyroglobulin to form T4 and T3. This action decreases thyroid hormone production. MMI also interferes with the conversion of T4 to T3. Since T3 is more potent than T4, this also reduces the activity of thyroid hormones (1, 2). The FDA approved MMI in March 1999 (3).

But MMI may cause side effects such as nephritis, liver cirosis, skin irritation, allergies and pharyngitis with fever (4). Thus, accurate determination of the drug by using simple and fast methods is a requirement in the fields of pharmaceuticals, nutrition, as well as clinical chemistry. 

Several analytical procedures have been described for the determination of MMI including thin layer chromatography (5), coulometry (6), conductometry (7), high-performance liquid chromatography with ultraviolet detection (8), spectroscopy (9-11), potentiometry (12), liquid chromatography with amperometric detection (13), and capillary zone electrophoresis (14).

Electrochemical methods have shown great advantages in pharmaceutical analysis due to their simplicity, fast response, and good sensitivity (15, 16). Moreover, the use of chemically modified electrodes is widely reported for sensitive and selective determination of various pharmaceuticals. The modified electrodes for the determination of MMI contain acetylene black/chitosan film (17), multi-walled carbon nanotubes (MWCNTs) (18), MWCNTs /electro-copolymerized cobalt nanoparticles-poly (pivalic acid) composite film (19) and carbon paste electrodes modified with Schiff base complexes of vanadium and cobalt (20, 21), nanocomposite of CdS NPs-RGO/IL (22) and MWCNTs - titanium dioxide nanoparticles (23).

Glassy carbon electrode (GCE) is one of the most common working electrodes used in electrochemical research (24). The electrode modifications and pretreatments have been widely used to improve the electrochemical responses of biological compounds and to construct electrochemical detectors. Among them, electrochemical pretreatment of glassy carbon electrode (EPGCE) seems to be a simple and less time consuming strategy in comparison to other procedures. For example, EPGCE has been applied in the determination of metal ions such as copper (25) and manganese (26), some organic molecules such as vitamin B_2_ (27), morphine (28), uric acid and epinephrine (29). In addition, EPGCE has also been used in the detection of biomolecules, such as DNA (30, 31) and extracted alkaloids (32). 

In this work, a simple and fast method for the accurate and precise determination of MMI in pharmaceutical preparations was developed using an EPGCE. The effect of experimental parameters on the electrode response was investigated. The electrode responded to micromolar concentrations of MMI at the optimized conditions. 

## Experimental

*Reagents and solutions*

Methimazole powder was a gift from Bakhtar Bioshimi Pharmaceutical Company (Kermanshah, Iran). Its stock solution (0.02 M) was prepared freshly before use. All other chemicals were of analytical reagent grade from Merck (Darmstadt, Germany) and were used without further purification. Thyromazole tablets were from Alhavi Pharmaceutical Company (Tehran, Iran). Doubly distilled water was used for the preparation of all solutions. Phosphate buffer solution (PBS, 0.1 M) was used as the supporting electrolyte and prepared by mixing different volumes of Na_2_HPO_4_ (0.1 M) and NaH_2_PO_4_ (0.1 M) solutions.

*Apparatus*

Electrochemical measurements were performed using a μ-Autolab (type III) potentiostat/galvanostat instrument. The data acquisition was performed using software NOVA 1.8. The three-electrode system consisted of a saturated calomel electrode (SCE) as the reference, a platinum wire as the counter electrode, and an electrochemically pretreated glassy carbon electrode (EPGCE) as working electrode. All experiments were conducted at room temperature. A pH meter (Jenway, Model 140) with a combined glass electrode was used to control the pH of the solutions.

**Scheme 2:**
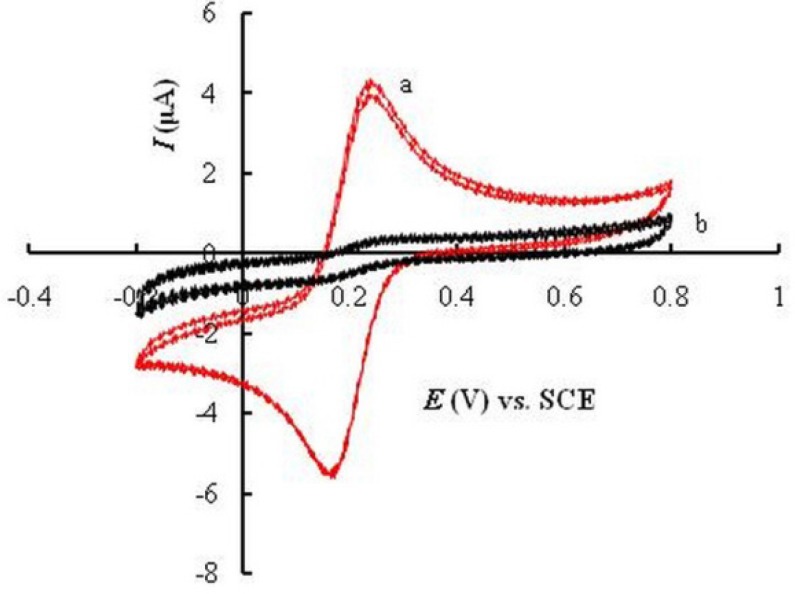
Mechanism of oxidation of MMI.

**Figure 1:**
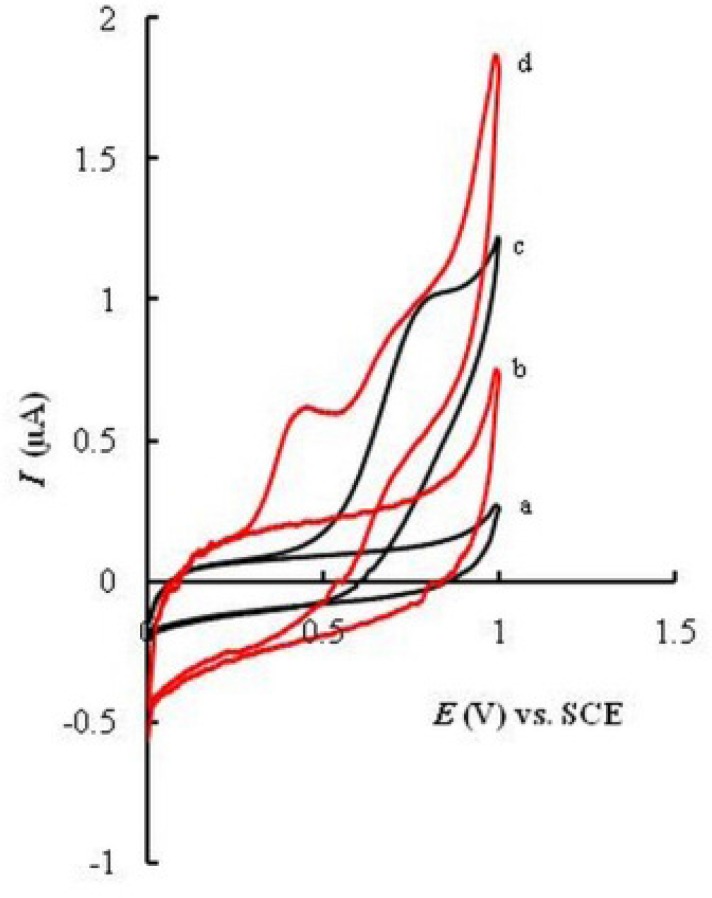
CVs of 0.1 mM K_3_Fe(CN)_6_ in 0.1 M KCl at UGCE (a) and EPGCE (b). Scan rate, 50 mVs^−1^.

**Figure 2:**
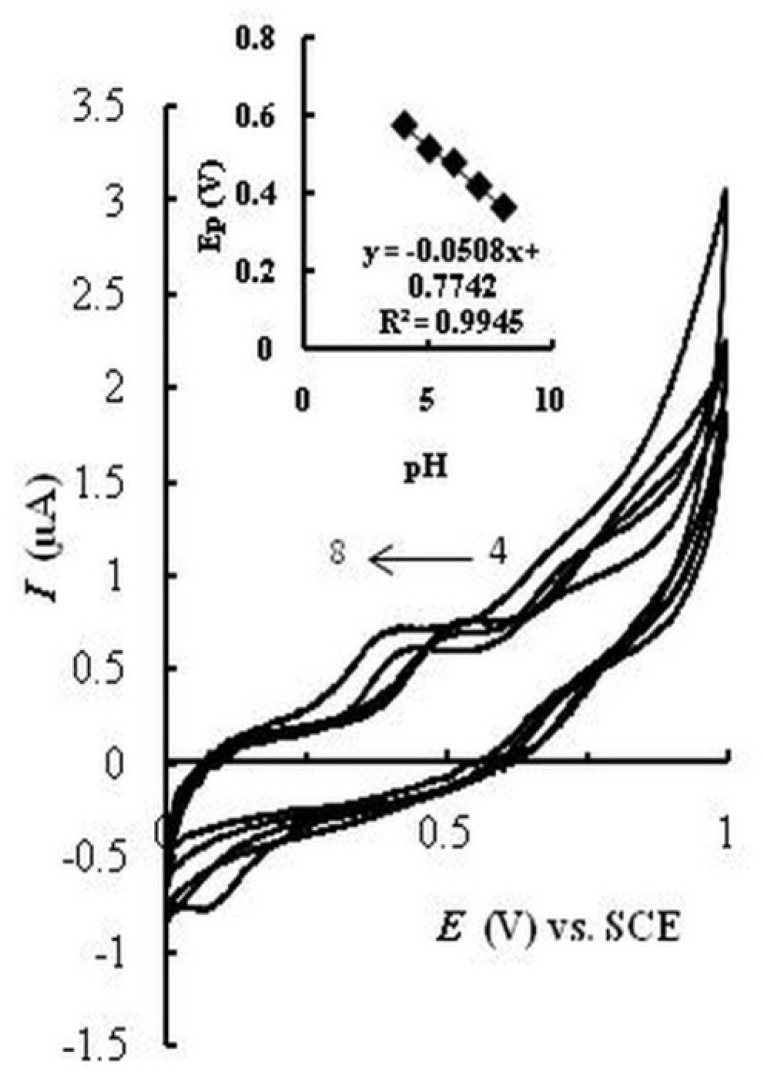
Cyclic voltammograms of MMI (10 μM) at the surface of UGCE (c) and EPGCE (d). Curves a and b show CVs for UGCE and EPGCE, respectively, in supporting electrolyte (PBS pH 7.5). Scan rate 50 mV s−1

**Figure 3:**
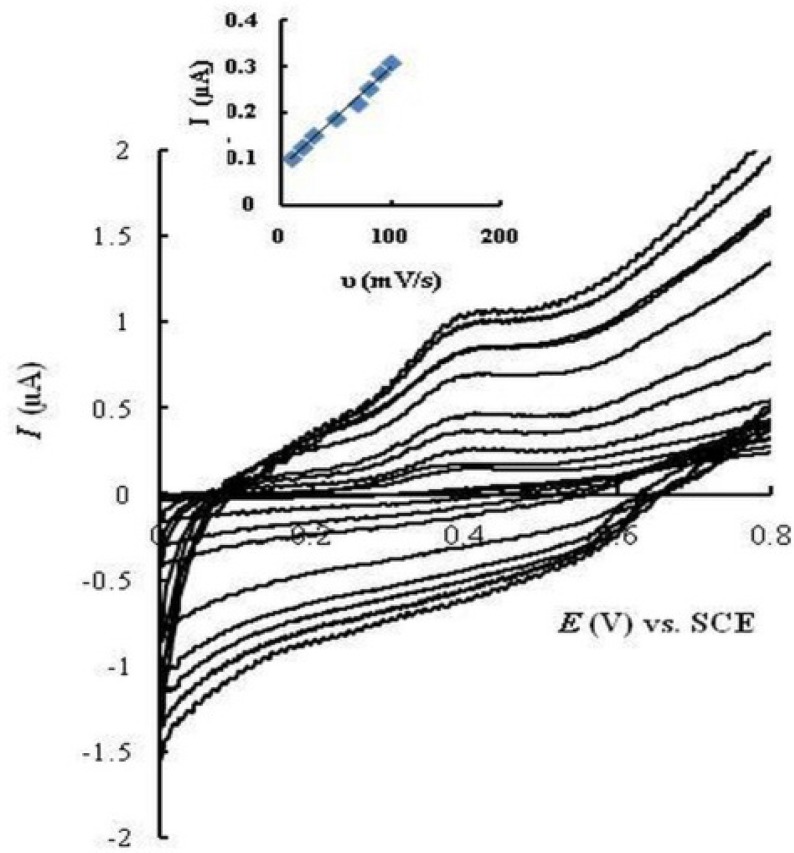
Cyclic voltammograms of MMI (10 μM) on EPGCE at various pHs (4-8). Inset: Plot of Ep against pH

**Figure 4:**
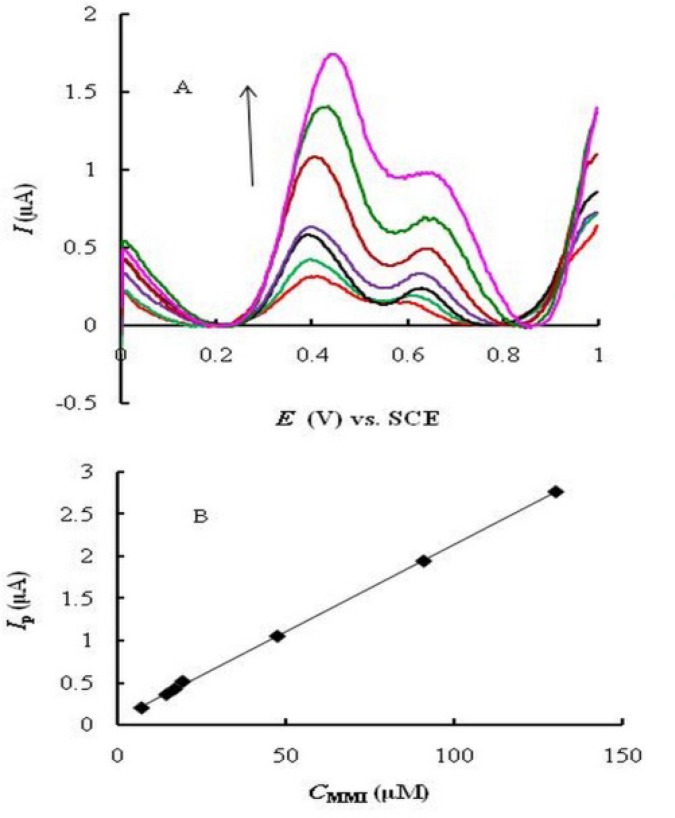
Cyclic voltammograms of MMI (20 μM) on PGCE at different scan rates (from inner to outer: 2, 10, 20, 30, 50,70,80, 90 and 100 mV/s, respectively).

**Figure 5:**
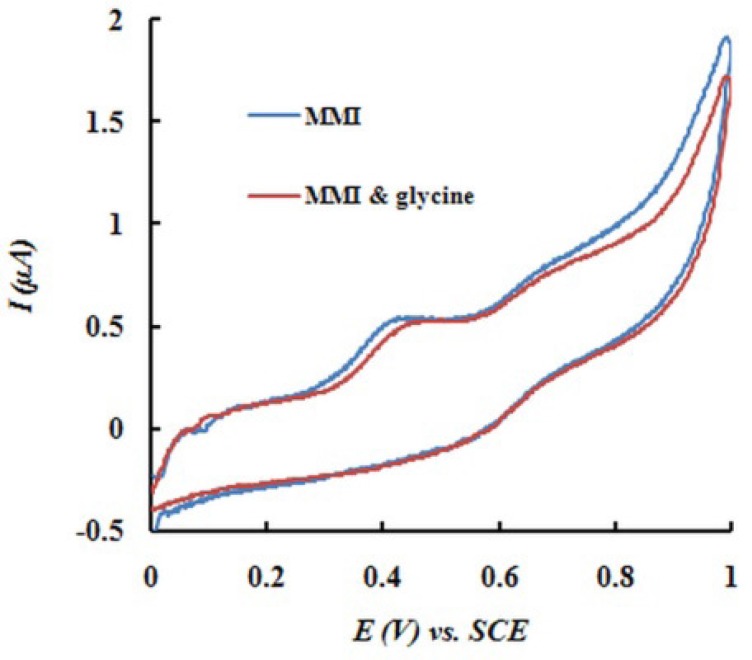
SWVs at EPGCE for various concentrations of MMI (A); Calibration plot (B). SWV characteristics: ΔEp = 20 mV; ΔEs = 5 mV; tp = 0.1 s.

**Figure 6:**
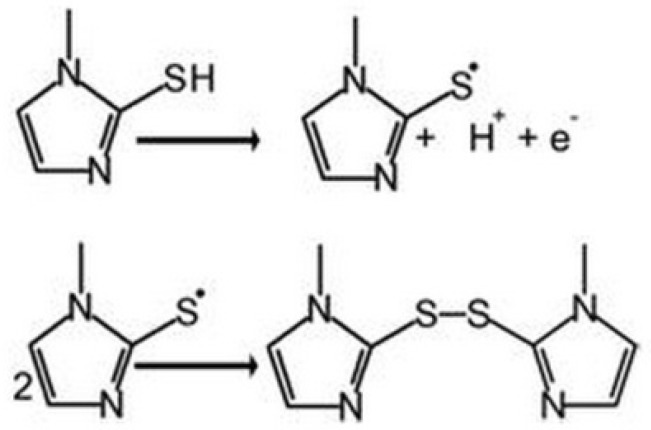
Cyclic voltamograms of MMI (0.1 mM) in the absence and presence of glycine (0.08 M). Scan rate 50 mV s−1, pH 7.

*Pretreatment of the glassy carbon electrode*

GCE was polished carefully with alumina powder of different particle sizes (1.0, 0.3 and 0.05 µm) and then rinsed with distilled water to remove residual particles. The polished electrode was electrochemically pretreated following a previously reported procedure (29, 30). Briefly, anodic oxidation at +1.75 V (vs. SCE) was applied to the electrode for 5 min in PBS (0.1 M, pH 7.0). The potential was then scanned between +0.3 and +1.3 V several times (20 scans). 

The electrode surface was cleaned by running a linear sweep in the reverse direction (33). The electrode was stored in doubly distilled water at room temperature when it was not in use.

*Voltammetric assay of MMI in samples of thyromazol tablets*

Ten thyromazol tablets (5 mg/tablet) were weighed and crushed to a fine powder in a mortar. A mass of powder equivalent to one tablet was dissolved in 25 mL of PBS (0.1 M, pH 7.5). It was introduced to an ultrasonic bath for 5 min, filtered and diluted with the same buffer in a calibrated volumetric flask (50 mL). Methimazole content of the formulation was determined by square wave voltammetric method (SWV) using EPGCE as the working electrode.

## Results and discussion

*Characterization of the electrochemically pretreated glassy carbon electrode (EPGCE)*

In order to confirm the effectiveness of anodization and potential cycling on the surface properties of GCE, cyclic voltammetric behavior of K_3_Fe(CN)_6_ was compared at the surface of untreated glassy carbon electrode (UGCE), with that at EPGCE. At UGCE, a quasi-reversible redox pair was observed for Fe(III)/Fe(II) (Figure 1a). The potential separation between two peaks (Δ*E*_p _= *E*pa - *E*pc) was about 76 mV and the ratio of peak currents (*I*_Pc _/ *I*_Pa_) was about unity. At the surface of EPGCE (Figure 1b), however, a large decrease of current was observed for the redox peaks of K_3_Fe(CN)_6_. This observation is attributed to the development of oxygen-containing groups at EPGCE during the electrochemical pretreatment of the electrode (32, 34, 35).

*Voltammetric behavior of methimazole on EPGCE*

Cyclic voltammograms were recorded for UGCE and EPGCE (Fig. 2) for MMI in buffer solution (PBS, 0.1 M, pH 7.5). The background current of EPGCE is higher than that of UGCE, indicating the buildup of phenolic and carbonyl oxygens during the pretreatment procedure (36). Figure 2 also depicts the cyclic voltammograms of MMI (10 μM) in the same buffer at the surface of UGCE and EPGCE. Due to the presence of –SH group, MMI had voltammetric signal at bare electrode through anodic oxidation. An irreversible broad anodic peak appeared at UGCE (curve c) with the anodic peak potential (*E*_pa_) of about 0.7 V vs. SCE. The mechanism of oxidation of MMI is shown in Scheme 2 (21):

Direct electrooxidation of -SH group is generally hampered due to the large anodic overpotential. However, at the surface of EPGCE, MMI exhibited a well-defined anodic peak (curve d) with the oxidation peak potential at about 0.4 V. Compared to UGCE, a large negative shift in oxidation potential (300 mV) is observed. 

This observation clearly proves the catalytic effect of EPGCE on the oxidation of MMI, as was reported in literature for other compounds (37, 38). The proton-coupled redox reactions (such as oxidation of MMI, Scheme 2) are believed to be catalyzed by the phenolic groups produced in a large amount at the EPGCE (39).

*Effect of pH*

The effect of pH on the current and potential of the catalyzed oxidation peak of MMI at EPGCE was studied in the pH range 4.0–8.0. Figure 3 represents cyclic voltammograms in acetate buffer solution (0.1 M, pH 4 and 5) and PBS (0.1 M, pH 6, 7, and 8) containing MMI. A negative shift in *E*_pa_ is observed with increasing pH from 4 to 8, which indicates the participation of protons in the oxidation of MMI. A linear relationship (*E*_pa_/pH) with a slope of about -51 mV (Inset) was obtained. The oxidation peak current increased with increasing pH and reached a maximum value at pH 7.5. Therefore pH = 7.5 was selected as optimized pH value for subsequent investigations.

*Effect of scan rate*

Cyclic voltammograms of MMI (20 μM) were recorded on EPGCE in PBS (0.1 M, pH 7.5) at various potential scan rates from 10 to 100 mV/s (Fig. 4). The oxidation peak current of MMI (*I*_pa_) increased linearly with scan rates (R² = 0.993), which shows adsorption of MMI at the electrode surface.

*Linear range, limit of detection and limit of quantitaion*

Square wave voltammetry (SWV) was used for the determination of MMI due to its higher speed and sensitivity compared to cyclic voltammetry. Two oxidation peaks were observed (Fig. 5A) corresponding to the catalytic (at 0.4 V) and diffusion (at about 0.65 V) currents of MMI. The catalytic peak current is substantially larger than the diffusion one. Under the optimized experimental conditions, the peak currents (at 0.4 V) were linearly proportional to MMI concentration in the range of 7.0^ _ ^130 μM (Fig. 5B) with a regression equation *I*p (A) = 0.020 *C*_MMI_ (M) + 0.067 (R^2^ = 0.9992). The limit of detection (LOD, based on S/N = 3) and limit of quantitation (LOQ, based on S/N = 10) were calculated to be 3.7 and 12.35 μM of MMI, respectively.

A comparison was made between the analytical characteristics of the proposed electrode with the previously reported modified electrodes (Table 1). The sensitivity of the method is comparable with MWCNTs – modified electrode (18) and superior to acetylene black/chitosan/GCE (17). Although LOD of the proposed method is higher than the other voltammetric methods reported in Table 1, but it was satisfactory in the analysis of real samples used in this study (Thyromazole tablets). Moreover, the preparation of the electrode was much simpler and very fast compared to the chemically modified electrodes reported; therefore, it could be prepared in a few minutes for each assay without the need for electrode storage.

*Interference studies*

The applicability of EPGCE for the voltammetric determination of MMI in the presence of potential interferents was studied. In this study, the peak current of MMI was recorded (Ip_1_). An excess amount of the potentially interferent species was added to the mixture and SWV was recorded (Ip_2_). If the change in peak current was less than ±5% (Ip_1_/Ip_2_ = 95-105%), the interference was not significant, otherwise, the concentration of the suspicious compound was decreased.

The potential interferences were chosen with respect to the real sample ingredients. Experimental results indicated that common ions, such as Na^+^, K^+^, Ca^2+^, Mg^2+^, and CO_3_^2−^ had no interference on the oxidation peak current of MMI. 80-fold concentration of glycine and 80-fold concentration of glucose did not disturb the determination of MMI (0.1 mM). Figure 6 shows typical cyclic voltammograms of MMI in the absence and presence of glycine (0.08 M). 

*Analytical applications*

A sample solution of thyromazole tablets (5 mg/tablet) was prepared as described in Experimental section. The SWV analysis of thyromazol tablets was performed using EPGCE and standard addition technique. Compared to the labeled amount of MMI, a recovery of 97.36 % was obtained. The relative standard deviation (RSD%) of the measurement for the samples was 1.33% (5 replicate measurements). The results show the applicability of EPGCE in pharmaceutical analysis with acceptable accuracy and precision. The electrode can be simply prepared only by applying potential for a few minutes. Therefore, there is no need for storing the electrode in certain conditions.

## Conclusion

Development of fast and easy procedures for drug analysis is desirable in pharmaceutical industries. In this work, an electrochemically pretreated glassy carbon electrode was used for the determination of methimazole. The anodic peak of methimazole shifted dramatically to less positive potentials at the surface of the pretreated glassy carbon electrode, compared to unmodified electrode. The catalytic effect was attributed to the appearance of phenolic and carbonyl groups at the electrode surface during the pretreatment step. These groups have catalytic effect on electron transfer mechanisms involving proton transfer. The electrode was used in the determination of methimazole by square wave voltammetry. The peak current of methimazole was linearly proportional to methimazole concentration in the range of 7.0-130 μM with a limit of detection of 3.7 μM. The electrode was successfully applied to the analysis of thyromazole tablets with a recovery of 97.36%. 
